# Vesicular Rash Following Immunisation With BTN162b2 Messenger RNA (mRNA) COVID-19 Vaccine: Vaccine Related or Coincidence?

**DOI:** 10.7759/cureus.22133

**Published:** 2022-02-11

**Authors:** Mohd Radzniwan A. Rashid, Sharifah Najwa Syed Mohamad, Anu Suria, Rokiah Shahra

**Affiliations:** 1 Department of Primary Healthcare, Faculty of Medicine and Health Sciences, Universiti Sains Islam Malaysia, Nilai, MYS; 2 Department of Family Medicine, Poliklinik Kesihatan, Universiti Sains Islam Malaysia (USIM) Healthcare, Nilai, MYS

**Keywords:** covid-19 vaccine, adverse events, vesicular rash, covid-19, post-vaccine rash

## Abstract

The introduction of the COVID-19 vaccines has led to an immense sense of relief for the global population. However, since the introduction of these vaccines, there have been several reports related to the side effects. A young woman presented to her primary care doctor with a vesicular rash three days after the BNT162b2 messenger RNA (mRNA) Pfizer-BioNTech vaccine, which was preceded by a low-grade fever for one day. Our case report highlights the challenges in diagnosing a vesicular rash post the BNT162b2 mRNA vaccine. Identifying the cause of a vesicular rash following vaccination has remained a challenge among primary care practitioners.

## Introduction

COVID-19 virus struck the world at the end of 2019. The virus has imposed a dramatic impact on health and has caused significant morbidity and mortality. The advent of vaccines in late 2020 helped to curb transmission and reduce the severity of the COVID-19 infections [1-3]. This is because the vaccines' efficacy was reported to be in the ranges of 86%-100% [4]. We have observed increasing cases of adverse effects being reported directly and indirectly post-vaccine inoculation. This case is focused on the BNT162b2 vaccine which is a lipid nanoparticle-formulated, nucleoside-modified RNA vaccine. Commonly reported adverse events following immunisations (AEFIs) with the BNT162b2 mRNA vaccine are self-limiting symptoms such as pyrexia, headache, arthralgia, nausea and injection site pain. However, anaphylaxis, myocarditis and skin manifestations cannot be ignored. The BNT162b2 mRNA vaccine is efficacious against coronavirus disease 2019 (COVID-19) with an effectiveness of up to 95%. It was approved as an option in the mass vaccination programme among other available types of vaccines against COVID-19. The case report illustrates how a vesicular rash appearing post BNT162b2 mRNA vaccine inoculation presented as a diagnostic challenge to her general practitioner.

## Case presentation

A 25-year-old woman was seen by her general practitioner with the complaint of vesicular rash three days after receiving her first dose of BNT162b2 (Pfizer-BioNTech) vaccination. It was preceded by a low-grade fever one-day post-vaccination. She claimed that a few rashes appeared gradually and scattered over her limbs, thigh and buttock. The rashes were described as itchy and painful. There were no coryza symptoms, insect bites or recent exposure to a varicella infection. She also never had chickenpox during her childhood. There was no history of close contact with any COVID-19 patients, and neither did she have other typical symptoms of COVID-19 infection such as sore throat, other upper respiratory tract symptoms or recent travelling history. Other than that, she reported having a history of mild allergy to strawberry preservatives, which when exposed, will be coupled with an itchy rash. She, however, did not experience any allergy to the natural fruit itself. In addition, there was no history suggesting that she was immunocompromised. She was not on any medication. She was worried about the rash as she was scheduled for her second dose in three weeks’ time.

**Figure 1:**
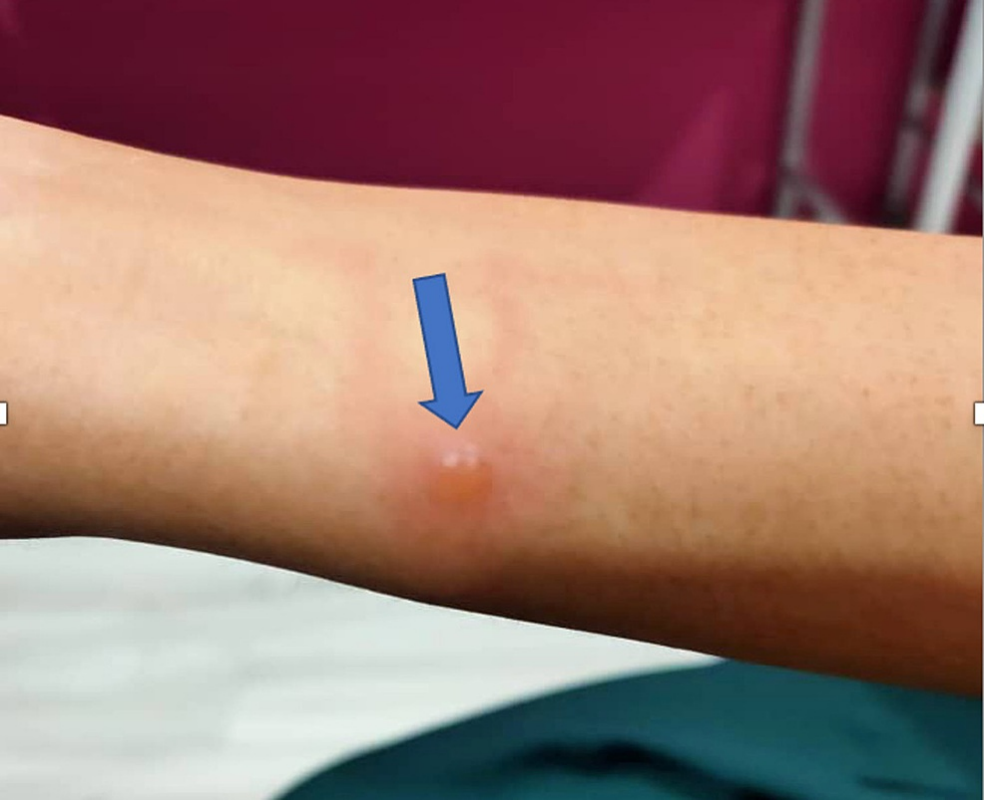
Vesicular rash on patient's right upper limb.

Clinically, she was not pale, with a temperature of 37.2°C, blood pressure of 120/80mmHg, and pulse rate of 80 beats per minute. There were scattered solitary vesicular rashes, varying in sizes, noted over her bilateral upper limbs on the anterior forearms, anterior thighs and buttocks. It was not specific to any dermatomal region. There was no rash on the palms, the soles or the mucous membranes. No Koplik spots were noted intraorally. Her neurological examination was normal. A picture of the rash on her upper limbs can be seen in Figure 1.

Blood was sent for a full blood count and varicella virus serology. The full blood count showed normal results, while the serology was reported as negative for varicella IgM and borderline for IgG. She was informed that she did not have an acute varicella infection and symptomatic treatment was subsequently given. Her symptoms were reported as an AEFI through the National Pharmaceutical Regulatory Agency (NPRA) of Malaysia. The rash and pruritus resolved in a week and she was allowed to receive her second dose in view that this was considered a minor adverse event. However, she developed a similar rash upon receiving her second dose of the same vaccine. Again, AEFI was subsequently reported. Fortunately, the rashes resolved spontaneously with symptomatic treatment and monitoring a week later.

## Discussion

Vaccine for COVID-19 has painted a new development in the profile of COVID-19 cases throughout the world. We have observed a significant decrease in morbidity and mortality caused by the virus with the implementation of mass inoculation of the COVID-19 vaccine in some countries. Nonetheless, the adverse effect as a result of vaccination still triggers great fear among humans. In fact, this contributes to lower uptake of vaccination in some countries and serves as ammunition for vaccine hesitancy groups to stand up for their vaccination refusal, making herd immunity much more difficult.

Occurrence skin manifestation post-vaccination was still variable at the time this case was reported. In fact, it was discovered during the clinical trial phase when 0.2% or less of Moderna’s vaccinated cohort developed rashes, including allergic, atopic and contact dermatitis; eczema; exfoliative rash; hypersensitivity reactions; injection site urticaria; papular urticaria; and vesicular rash, among others [5]. While clinical trial results for BNT162b2 mRNA COVID-19 vaccine reported mild-to-moderate pain at the injection site within seven days after administration, with severe pain in <1% of participants and redness or swelling in a lower percentage. Local reactions incidence did not increase after the second dose and was mostly mild-to-moderate and resolved within one to two days [6]. Another study reported that of those who had a cutaneous reaction to the first dose, 95% received a second dose, and 83% had no recurrent episodes at the second dose. Contrarily, among those who had no reaction at the first dose, 2.3% reported having the more common rash and itching post-second dose [7]. Additionally, a case report in Turkey had reported that the development of shingles post COVID-19 vaccine was also possible [8].

Much literature has reported that the skin lesion post-COVID-19 vaccination is not problematic. For example, an Italian single-centre case series observed those skin conditions that appeared post BNT162b2 mRNA COVID-19 did not constitute a contraindication to the second dose of vaccine as many were transient and self-limiting [9]. Therefore, the mild nature of cutaneous manifestation post-COVID-19 vaccination should be counselled and explained carefully to a patient prior to the administration of the vaccine in order to alleviate any worrying thoughts. This is as opposed to other serious adverse reactions such as severe anaphylactic reaction and myocarditis, which has been implicated by the Pfizer vaccine, while the risk of thromboembolism was noted with the AstraZeneca vaccine [10-13].

It is still of utmost importance not to assume that the symptoms were due to the vaccine without excluding other possible causes of vesicular rash and fever. In the above case, it was necessary to exclude varicella infection as a possible cause for her symptoms. However, there were no successive crops of the vesicular lesion and there was no history of contact within 10-21 days prior which was a typical incubation period for varicella infection [14]. The fever and malaise she experienced prior to the rash eruption could be attributed to the common side effects of the vaccine itself [15].

Cutaneous manifestation of COVID-19 infection could be considered a differential in this case. Some studies have reported that among the skin manifestation of COVID-19 are vesicular rash, urticaria, maculopapular rash, chilblains, purpura and more [16,17]. However, in this patient’s case, though it was thought to be a differential diagnosis, the COVID-19 test was not done. Owing to the lack of other typical and specific symptoms of COVID-19 such as dry cough, muscle weakness, and chest pain subjects the diagnosis of COVID-19 infection is less likely [18]. In addition to that, she was also not in close contact with a COVID-19 index, which further out rules the diagnosis.

Drug reaction is another possibility, i.e. drug-induced rash. ICD 10 has classified this as L27.0 - Generalised skin eruption due to drugs and medicaments taken internally [19]. Other causes of blisters needed attention. For example, Steven Johnson Syndrome needs to be thought in mind as this life-threatening condition requires intensive care and management. Nonetheless, for the diagnosis to be made, the progression should be acute and involvement of systemic reaction should be present, where this is not apparent in the above case. The results only showed borderline positive for IgG which may indicate past infection [20]. The fact that a similar rash appeared post-second dose, had made the diagnosis related to the varicella infection less likely. Therefore it could be triggered by the BNT162b2 mRNA (Pfizer-BioNTech) vaccine-related side effect, i.e. in accordance with ICD 10. The case has been reported as AEFI so that the authorities could conduct a proper investigation and document it for national statistics on COVID-19 vaccine-related AEFI. Importantly, while managing such skin lesion post-COVID-19 vaccination, the patient is counselled that it is usually self-limiting and not life-threatening.

## Conclusions

Rash eruption post-COVID-19 vaccination is not very common and fortunately, most are benign. However, when this happens, it should be managed accordingly to avoid any serious and life-threatening conditions because it may be the first sign of an anaphylaxis reaction. Careful observation and considerations play an important role. Patient empowerment about the nature of the problem and safety netting advice is also crucial in order to identify any progressing symptoms and signs that require further attention and management from healthcare providers.
